# Supplementary figures and images for: Genome-Wide Identification of the GhANN Gene Family and Functional Validation of GhANN11 and GhANN4 under Abiotic Stress
Source: Int J Mol Sci. 2024 Feb 4;25(3):1877. doi: 10.3390/ijms25031877 (PMC10855742; doi:10.3390/ijms25031877)

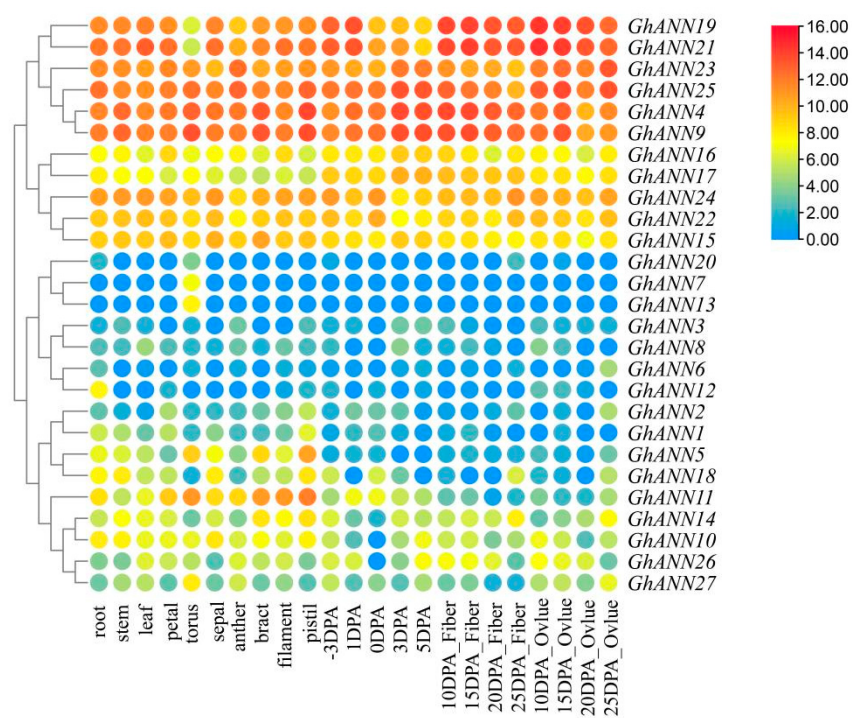

**Figure S1.** The *GhANN* gene expression profiles in different tissues.

Supplement: Supplementary file 1 [file ijms-25-01877-s001.zip › Figure S1.pdf]
